# Microencapsulation of Capsaicin in Chitosan Microcapsules: Characterization, Release Behavior, and Pesticidal Properties against *Tribolium castaneum* (Herbst)

**DOI:** 10.3390/insects14010027

**Published:** 2022-12-27

**Authors:** Su-Fen Cui, Jin-Wei Wang, Hai-Feng Li, Ran Fang, Xin Yu, Yu-Jie Lu

**Affiliations:** School of Grain Science and Technology, Jiangsu University of Science and Technology, Zhenjiang 212004, China

**Keywords:** capsaicin, chitosan, microcapsule, *Tribolium castaneum*, integrated pest control

## Abstract

**Simple Summary:**

Grains are a major food resource for humans and ensuring food safety is essential. However, stored grains can be heavily infested by insect pests, leading to serious losses. Traditionally, fumigation based on chemical insecticides has been used for pest control, which include broad spectrum insecticides with high insecticidal efficiency. Unfortunately, this leads to food contamination and environmental pollution. Moreover, insects have evolved a higher resistance to these pesticides after long-term exposure. Developing new alternative insecticides has become important work in the field of grain storage. In this paper, we prepared a microcapsule using capsaicin, chitosan, and carboxymethyl chitosan, which has a high chemical stability and insecticide activity. Modern analytical methods such as FTIR, XRD, and SEM were used to determine these materials’ morphologies, crystal structure, and components, revealing that the particles had an amorphous nature, a uniform size, good adhesion, and significant slow-release characteristics. *Tribolium castaneum* (Herbst), an important pest insect, was selected as the experimental object. The results showed that this material had a higher insecticidal efficiency than pure capsaicin, both in inhibiting larvae development and in distorting the normal adult insect reproduction, revealing that this microcapsule has application potential as an insecticide for the insect control of stored products.

**Abstract:**

Capsaicin is a capsaicinoid in hot chili peppers, with excellent antibacterial and antimicrobial activities and a good safety profile, but its poor solubility and instability restrict its effectiveness. This limitation may be mitigated by encapsulation. Herein, capsaicin microcapsules (CCMs) were prepared through layer-by-layer self-assembly, using chitosan and carboxymethyl chitosan as shell materials. The chemical and microstructure structural characterization was evaluated by the methods of Fourier transform infrared spectroscopy (FTIR), scanning electron microscopy (SEM), and X-ray diffraction (XRD). The SEM indicated the microcapsules were irregular in shape with an average size of about 100 μm. The encapsulation had a high loading efficiency of 64.31%. FTIR and XRD revealed the absence of the interaction between the core and shell materials and the amorphous nature of the CCMs. The analysis results of the microcapsules’ release behavior showed the burst release of capsaicin in 7 days and a slow progression afterward in three solutions, with the highest release properties in a basic solution, followed by acidic and neutral salt solutions. The entomotoxicity of CCMs was conducted against *Tribolium castaneum* (Herbst), and its efficacy was compared with pure capsaicin. The CCMs were found to be highly effective against this pest. The LC_50_ value for capsaicin and its microcapsules was 31.37 and 29.75 mg/kg on adults, respectively. According to these values, *T. castaneum*’s development and reproduction were significantly inhibited compared with the control group. The excellent physicochemical characteristics and insecticidal performance show a high application value for integrated pest control.

## 1. Introduction

Stored products in subtropical and tropical locations are often damaged by insect pests during storage [1]. Worldwide, the losses of stored products caused by pests and mites have been estimated to be 5%~15%, and this rate could reach 10%~40% in some developing countries [2]. The amount of damage in quality and quantity when converted into monetary terms may reach one hundred billon USD [3]. The flour beetle, *Tribolium castaneum* (Herbst) (Coleoptera: Tenebrionidae) is a major secondary pest of stored grains and cereal products throughout the world, causing serious economic losses in warehouses and mills [4]. It is suggested to damage more than 9% of stored grains in developed counties, while causing more than 20% loss in developing countries due to its high fecundity rates, relative longevity, and strong resistance to various adversities [5]. Due to the magnitude of such losses, it has become a key target for prevention and treatment. 

Fumigation based on chemical insecticides is currently used for pest control, but this leads to food contamination and environmental pollution. Moreover, insects have evolved a higher resistance to these pesticides after long-term exposure [6,7,8]. Considering these disadvantages, these insecticides are not an environmentally sound approach for controlling the population of stored product insects. Recently, a growing number of natural oils have been reported to protect grain from insect damage, which are suggested as alternatives to the currently used fumigants [5]. Some plant secondary metabolites, such as alkaloids, flavonoids, terpenoids, and phenols, show insecticidal potential against stored product insects. Importantly, these natural extracts are highly effective, inexpensive, and safe for human beings and the environment [9].

Capsaicin (trans-8-methyl-N-vanilla-6-nonenylamide, CAP) is one of the major components of capsaicinoid from chili peppers and similar plants, with many bioactivities, such as being anti-inflammatory, antitumoral, antioxidant, anticarcinogenic, and antibacterial [10,11,12,13]. It has been widely used in the fields of food, medication, agriculture, and so on. Capsaicin naturally exudes a pungent odor, which can protect chili peppers from damage caused by pests (insects, microbes, or mice) [14,15]. Several studies have suggested that CAP can interfere with the foraging, food-averse migratory behavior, and social behavior of insects. For instance, the effects of CAP on mosquito vectors *Anopheles Stephensi* (Liston) (Diptera, Culicidae), *Sitophilus oryzae* (Linnaeus) (Coleoptera, Curculionidae), *Drosophila melanogaster* (Meigen) (Diptera, Drosophilidae)*,* and *Nilaparvata lugens* (Stal) (Hemiptera: Delphacidae) have been well documented [16,17,18]. These studies showed the potential application of CAP in pest control, although the underlying mechanism remains unknown.

Unfortunately, some disadvantages of CAP, such as its poor solubility, instability, and intensively pungent odor, limit its practical application. Firstly, although it can be absorbed rapidly in the intestine, oral delivery of CAP has a low bioavailability, due to its poor aqueous solubility (<0.1 mg/mL) and first-pass metabolism in the liver [19]. Secondly, its efficiency is short-lived due to its quick-release properties [20]. Thirdly, it causes strong irritation to the skin and eyes of operators during the operating process. Exposure to a high dose of CAP (>100 mg CAP per kg body weight per day) may be harmful, leading to breathing difficulties, convulsions, and even suffocation or death. Therefore, current studies are focusing on improving its properties by various methods. For instance, encapsulating it could mitigate these problems, [1]. The process of entrapping an active ingredient into the hollow cavity of a microcapsule is called microencapsulation. Encapsulation can improve the active ingredient’s properties through adjusting the particle size, structure, and polymer material with the proper type and proportion, according to the specific end-use. There have been several studies on the preparation process of encapsulation [21].

The preparation of microencapsulation using natural polymers such as polysaccharides as the coating material is of particular interest, due to their larger availability and higher biocompatibility [22]. Chitosan (deacetylated chitin), a cationic natural polymer, is well-known as a good coating material, with its unique features, such as biocompatibility, film-forming ability, biodegradability, and antimicrobial and antifungal activities [23,24]. It is widely found in the exoskeletons of arthropods, crustaceans, and fungal cell walls, and it has been approved by the U.S. Federal Drug Administration (FDA) as a safe material for biomedical, food, and plant protection [25]. However, the application of chitosan is limited because of its low dissolvability and beam focus on porosity. The solubility of chitosan could be improved by chemical modification; for example, some derivatives might be prepared by quaternarization or by introducing hydrophilic groups into the macromolecular chain of chitosan. Carboxymethyl chitosan (CMCS), an important kind of water-soluble chitosan derivative, is prepared by introducing carboxymethyl groups to create its ampholytic character, a higher biodegradability, and more possibilities for application [26]. The degree of carboxymethyl substitution in the CMCS depends on the ratio of amine to the reagent used and the reaction conditions. The relevant properties and applications in the preparation of microcapsules have been the focus of recent reviews [24,27,28,29].

Although the preparation of CAP microcapsules using chitosan as a shell material has already been reported, there is little information about its insecticidal effect against *T. castaneum* in stored grain [5,20]. In this study, the capsaicin/chitosan microcapsules (CCMs) containing CAP with chitosan and CMCS as shell materials were prepared by the complex coacervation method. The structural characteristics, release properties, and their insecticidal effect against *T. castaneum* were determined.

## 2. Materials and Methods

### 2.1. Materials

High-density chitosan (deacetylation degree ≥ 85%) and carboxymethyl chitosan (substitution degree ≥ 80%) were purchased from Shanghai McLean Biochemical Technology Co., Ltd. (Shanghai, China); capsaicin (99 wt%) was purchased from Hubei Xinrunde Chemical Co., Ltd. (Wuhan, China); polytetrafluoroethylene(58~62 wt%) was purchased from DuPont (Wilmington, Delaware, USA); the other reagents and chemicals used were purchased from Sinopharm Chemical Reagent Co., Ltd. (Shanghai, China).

### 2.2. Preparation of the CCMs

First, 10 g of chitosan was dissolved in 100 mL of acetic acid (1 wt%); then, 20 mL of phosphorous acid (0.5 g/mL) and 13 mL of formaldehyde (37%~40 wt%) were added into the solution and stirred well. The mixture was heated to 70 °C and maintained for 3 h under stirring at 400 rpm. When the reaction was completed, the solution was naturally cooled, alcohol deposited, suction filtered, and freeze dried to obtain the phosphorylated chitosan.

Capsaicin (0.1 g/mL) was prepared in ethanol, added into a carboxymethyl chitosan solution (0.01 g/mL) at the ratio of 1:1, and stirred for 30 min using a magnetic stirrer. Chitosan (5 mg/mL) was prepared in a HCl solution (0.1 mol/L) and added into the mixture above with the same volume, emulsifying the mixture for 20–30 min. After the reaction, the reaction solution was filtered, washed several times with distilled water, and then dried at 40 °C for 10 h to obtain the CCMs. The prepared CCMs were ground into powder and stored at 4 °C for further analysis.

### 2.3. Efficiency of the CCMs

A spectrophotometric method for the determination of capsaicin in the CCMs was studied. Different concentrations, 0, 40, 80, 120, 160, and 200 μg/mL, of the CAP solutions were separately prepared in an ethanol solution (50% v:v). All samples were stirred well under ultrasonic and filtered; then, the colorimetric assay at 280.5 nm was carried out for absorbency of the respective concentration using a UV spectrophotometer (UV-7504, Shanghai Xinmao Instrument Co., Ltd., Shanghai, China). Taking the consistency as the X-axis and the absorbency as the Y-axis, the standard curve was drawn.

About 0.01 g of CCM powder was dissolved in a 5 mL of ethanol solution (50% v:v) to make 25 mL. After it was completely mixed, the solution was filtered, and the absorbance value was determined at 280.5 nm by a UV spectrophotometer. According to the standard curve, the loading efficiency (LE) of the CAP of the CCMs was calculated using the following Equation (1).

(1) $$\mathrm{LE}\%=\frac{c\times v}{10^{6}\times m}\times 100\%$$

where *c* is the content of CAP in CCMs solution (μg/mL), *v* is the total volume of solution (mL), and *m* is the mass of CCMs used (g).

### 2.4. Release Effect of the CCMs

A certain amount of CAP was separately dissolved in sodium hydroxide solution (pH = 9.94), acetic acid solution (pH 3.03), and sodium chloride solution (5 wt%) to prepare a set of six standard solutions of 0, 4, 8, 12, 16, and 20 μg/mL in three sustained-release solutions; then, the absorbance was measured at 280.5 nm by UV spectrophotometer. The standard curve between the absorbance and CAP concentration in different sustained-release solutions was drawn.

Three samples of 0.030 g of CCMs were separately loaded into a dialysis bag (the molecular weight cutoff was 8000–14,000), sealed, and put into 100 mL of sodium hydroxide solution (pH 9.94), acetic acid solution (pH 3.03), and sodium chloride (5%) sustained-release solution. A certain volume solution was taken for colorimetric assay every 24 h (every 48 h after 7 d); meanwhile, we added the same volume of sustained-release solution to reach 100 mL. The release rate (ARR) of the CCMs was calculated using the following Equation (2):

(2) $$ARP=\frac{C\times 100+\sum W}{M\times C_{MC}\times 1000}\times 100\%$$

where *C* is the concentration of the CAP in the sustained-release solution (μg/mL), Σ*W* is the accumulated CAP content taken out during the colorimetric assay (μg), *M* is the total mass of the initial sample (100 mL in the test), and *C_MC_* is the percentage of the CAP in the CCMs.

### 2.5. Structural Characterization of the CCMs

A Fourier transform infrared spectrometer (FTIR) (Nicolet iS 20, Thermo Fisher Technologies Inc., Waltham, MA, USA) was used to determine the vibrational properties. The sample and KBr powder were mixed evenly, ground into powder, squeezed into a thin sheet, and placed into the sample holder; then, the FTIR spectra were recorded on an FTIF spectroscope in the 400–4000 cm^−1^ range at a resolution of 4 cm^−1^.

A scanning electron microscope (SEM) (Hitachi SU8010, Hitachi, Japan) was used to observe the morphological characteristics. The sample was attached to the sample stage by conductive adhesion with gold sputtering and observed by using SEM at 15 kV.

An X-ray diffractometer (Ultima IV, Nippon Science Co., Ltd., Tokyo, Japan) was used to examine the crystal structure. The X-ray source employed was a copper X-ray tube (CuKα, λ = 0.154056 Å), effective at 40 kV and 30 mA. The average particle size (D) was calculated using the Debye–Scherrer Equation (3) [30].

(3) $$D=\frac{K\times \lambda }{\beta \cos\theta }$$

In the formula, *k* is the Scherrer constant, which is generally 0.89; *λ* is the X-ray wavelength (CuKα, *λ* = 0.154056 Å). *β* is the full half-maximum width of the CCMs, and the unit is generally rad; *θ* is the diffraction angle.

### 2.6. Effect of the CCMs on T. castaneum

#### 2.6.1. Insects

*T. castaneum* were reared on whole wheat flour with 5% yeast (WWF) in an environmental chamber with 28 ± 2 °C and 65 ± 5% r.h. The insects were inbred in the lab by sib-mating for at least 10 generations; so, they were genetically and phenotypically stable. About 2–3-day-old adult flour beetles were selected for the following experiments.

#### 2.6.2. Mortality of *T. castaneum*

After starvation for 24 h, *T. castaneum* adults were treated with different concentrations of CCMs and CAP, respectively. Specifically, CCMs and CAP were separately prepared at five dose rates of 10, 20, 30, 40, and 50 mg/Kg WWF. Then, the flasks were shaken manually to mix the seeds well. Next, 20 unsexed adults were introduced into each flask with 5 g of feed above. The internal “necks” of these flasks were covered by polytetrafluoroethylene to prevent the insects from escaping. Lastly, we sealed the flasks with a plug to prevent gas from leakage. All bioassays were performed at 28 ± 2 °C and 65 ± 5% r.h during the experiment. Insect mortality was checked by counting the number of dead beetles in each treatment at regular intervals (24 h), while the LC_50_ value was calculated. Each treatment was replicated three times. Meanwhile, the control was carried out with WWF only. The adults that remained motionless under a stereo microscope (SZX9, Olympus, Delaware, Tokyo, Japan) after prodding each flask several times were considered dead [31,32].

#### 2.6.3. Growth and Development of *T. castaneum*

One hundred pairs of 2-day-old *T. castaneum* adults were introduced into a flask containing WWF for oviposition; then, they were removed 6 h later. These eggs were allowed to live in the original flask until they developed to fourth instar larvae. The staging into instars was based on the number of undergone ecdysis; in particular, the newly eggs laid were collected and checked daily to mark the time of hatching and each ecdysis [33].

The newly emergent fourth larvae were used for the following experiments. Two groups of 20 fourth instar larvae were selected; then, they were separately treated with CCMs and CAP during the entire experiment. Specifically, 5 g of WWF was placed in each flask; then, they were separately mixed with CCMs and CAP at the LC_50_ value. Following this, these flasks were shaken manually several times to achieve equal distributions of the materials with the WWF. After 24 h of starvation, 20 fourth instar larvae were introduced into each flask above. The internal “necks” of these flasks were covered by polytetrafluoroethylene to prevent the insects from escaping, and we sealed the flasks with a plug to prevent leakage of gas. The control was carried out with WWF only. All biological experiments were duplicated three times. The treated and control insects were examined once a day for survival larval development; the individuals that failed to emerge were considered dead. The numbers of pupae and adults that emerged in each flask were calculated, and then, the pupation and emergence rates were calculated, respectively. The new adults in each replication were weighed to calculate the average body weight. The total time of the developmental period of *T. castaneum* was determined by counting the days between the fourth instar larvae and adult emergence in each flask; then, the developmental time delay relative to the control was calculated. 

#### 2.6.4. Reproduction of *T. castaneum*

The whole flour powder feeds containing the LC_50_ of the CCMs and CAP were separately placed into each flask. Then, 40 two-day-old female and male adults were selected and separately transferred into these flasks with CCM and CAP diets after 24 h of starvation. Then, 96 h later, 10 pairs of surviving adults treated with the same pesticide (CCMs or CAP) were collected and transferred to a new flask with WWF only and allowed to oviposit and lay eggs. Meanwhile, the control was carried out in flasks with WWF. All biological experiments were repeated three times. The treated and control groups were examined. The number of newly emergent eggs, as well as the hatching rate, were recorded successively for 7 days. 

### 2.7. Data Analysis

The mortality of insects in treatments was corrected according to the formula of Abbott(1925) [34] Equation (4).

(4) $$x=\frac{\mathrm{Mortality}\;\mathrm{in}\;\mathrm{treatments}\left(\%\right)-\mathrm{Mortality}\;\mathrm{in}\;\mathrm{control}\left(\%\right)}{100-\mathrm{Mortality}\;\mathrm{in}\;\mathrm{control}\;\left(\%\right)}\times 100$$

In the formula, *x* is the actual morality in treatments.

The lethal concentration to 50% of insects (LC_50_) and half knock-down time ( KT_50_) were obtained using probit analysis. Data analysis was performed using SPSS for Windows (Version 20.0; IBM Corp., Armonk, NY, USA). Data were analyzed using one-way analysis of variance (ANOVA) followed by Tukey’s multiple range test [31]. The results are expressed as the mean ± standard deviations (SD) and considered statistically significant at *p* < 0.05.

## 3. Results 

### 3.1. Loading Capacity and Release Behavior of the CCMs 

The amount of capsaicin encapsulated in particles was obtained from the standard curve of concentration versus its absorbency (Figure S1a). The linear equation was obtained (y = 0.366 x + 0.302, R^2^ = 0.9859), and the encapsulation efficiency of the CCMs prepared was 64.31%, which showed that this microcapsule was well microencapsulated with a high ratio of capsaicin.

**Figure 1:**
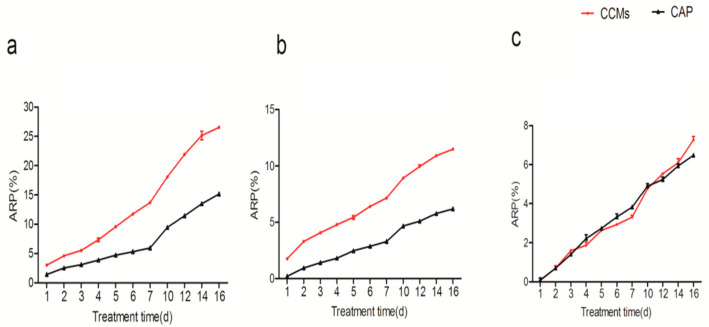
The sustained release properties of the CCMs synthesized in varied solutions: (**a**) NaOH solution (pH 9.94), (**b**) HAc solution (pH 3.03), and (**c**) NaCl solution (pH 7). Data were expressed as mean ± SD (*n* = 3); CAP, capsaicin; CCMs, capsaicin/chitosan microcapsules.

The concentration of CAP released from the CCMs in all three solutions (NaOH, HAc, and NaCl) increased with time (Figure 1). The amount of CAP was obtained from the standard curve of concentration versus its absorbency in the three solutions. The linear regression equations of these standard curves were as follows: in the NaOH solution: y = 0.0124 x + 0.0254, R² = 0.9983 (Figure S1b); in the HAc solution: y = 0.016 x + 0.0379, R² = 0.9979 (Figure S1c); in the NaCl solution: y = 0.0126 x + 0.032, R² = 0.9973 (Figure S1d), where x was the concentration of capsaicin, and y was the absorbency at 280.5 nm. The release rates of the CCMs and CAP were calculated according to Equation (2).

The prepared microcapsules showed the burst release of capsaicin in 7 days and a slow progression afterward in the three solutions, which was similar to that of the pure CAP (Figure 1). There was a significant difference in the three pH conditions, with the highest release rate in pH 9.94 (Figure 1a), followed by that in the pH 3.03 (Figure 1b) and pH 7.0 solutions (Figure 1c). The CAP in the loaded CCMs showed better release properties than that in the original state in the acid and basic solutions; although it showed no significant difference between the CCMs and CAP in the 10-day exposure in a neutral solution, the CCMs showed a higher release rate than the CAP afterward (df = 21, F = 1059.67, *p* < 0.05).

### 3.2. Structure Characteristics of the CCMs

**Figure 2:**
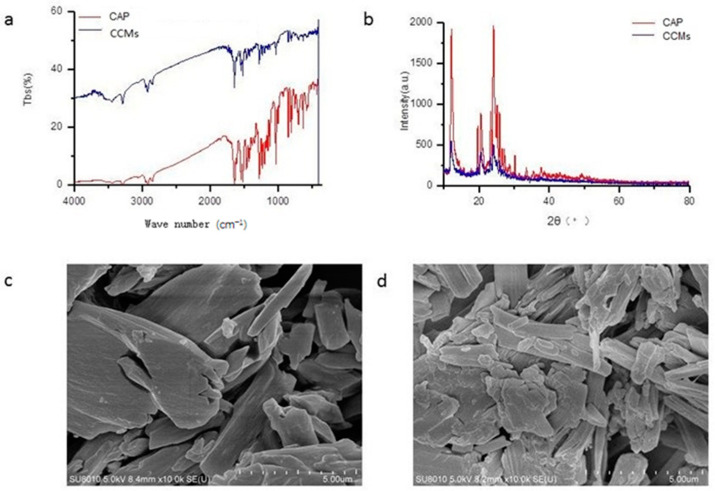
Biophysical characterization of the synthesized CCMs. (**a**) Fourier infrared spectra at different wavelengths; (**b**) the XRD patterns; (**c**,**d**) SEM image of the CAP and CCMs. CAP, capsaicin; CCMs, capsaicin/chitosan microcapsules.

The FTIR spectra of the CAP and CCMs is shown in Figure 2a. In the CAP, the spectrum showed prominent peaks at 3293.43, 2926.4, 1641.9, 1524.6, 1469.5, 1279.73, and 1033.9 cm^−1^. The spectrum of the CCMs was similar to that of the CAP, with characteristic absorption peaks appearing at 3292.46, 2925.48, 1641.8, 1523.49, 1469.73, 1278.73, and 1034.67 cm^−1^. In addition, the absorption peak intensity of the CCMs was much weaker as compared to the CAP (Figure 2a).

The crystal structure and particle size of the microcapsules were further identified by XRD (Figure 2b). The XRD graph of the CAP reflected the strong diffractions (2θ) at 12.080°, 20.467°, 24.152°, 25.162°, and 30.369°. The XRD graph of the CCMs showed 2θ = 12.439°, 20.032°, 24.139°, 25.120°, and 30.439°. However, some characteristic peaks of chitosan and CAP were weakened or not present in the spectrum of the CCM. The average particle size of the CCMs calculated, according to formula (2), was 106.24 μm. The crystallite microcapsules size obtained by XRD analysis was confirmed again by SEM. The morphology and distribution of the CAP and CCMs were investigated by SEM (Figure 2c,d). The micrographs showed the changes in the CAP that occurred during the preparation of the CCMs. The capsaicin particles were amorphous in shape, while the CCM particles formed a similar shape with a much smoother surface. The average size of the CCM granules was about 100 μm.

### 3.3. Effect of the CCMs on the Mortality of T. castaneum

**Figure 3:**
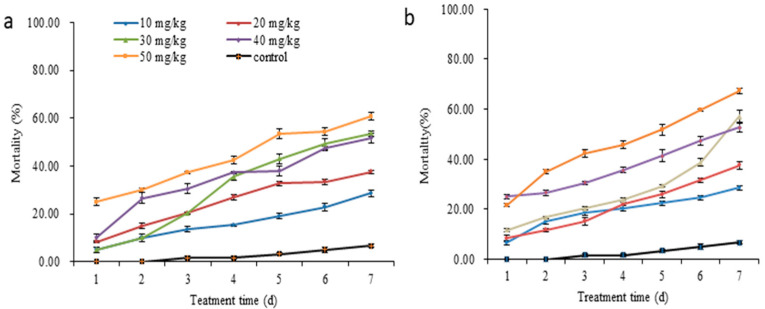
Changes in the mortality of the *T. castaneum* treated with CCMs (**a**) and CAP (**b**) at different concentrations. Data are expressed as mean ± SD (*n* = 3). CAP, capsaicin; CCMs, capsaicin/chitosan microcapsules.

The mortality of the *T. castaneum* adults treated with CCMs and CAP was calculated, which showed a time- and concentration-dependent manner (Figure 3; Table 1). The mortality increased significantly with the exposure time increased at a certain concentration of CCMs and CAP. The mortality increased significantly with increasing concentration of CCMs and CAP. When *T. castaneum* adults treated for 7 days, the toxicity regression equations of the CAP and CCMs obtained were: CAP, y = 1.196 x − 1.789, *p* < 0.001; CCMs, y = 1.011 x − 1.989, *p* < 0.05; where y was the mortality (%) in probits, and x was the log_10_ dose. Based on these equations, the LC_50_ of CAP and CCMs were 31.37 and 29.75 mg/kg on day 7, respectively (Table S1). When *T. castaneum* adults treated at 30 mg/kg CAP or CAP for increasing days, the mortality increased significantly. The KT_50_ regression equations of the CAP and CCMs obtained were: CAP, y = 2.404 x − 1.846, *p* < 0.001; CCMs, y = 1.442 x − 1.376, *p* < 0.001; where y was the mortality (%) in probits, and x was the log_10_ time. The Kt_50_ of CAP and CCMs were 5.86 days and 9.00 days at the concentration of 30 mg/kg, respectively (Table S2).

### 3.4. Effect of the CCMs on the Development of T. castaneum

The growth and development of the *T. castaneum* larvae were affected by the CAP and CCMs (Table 1). The development time to reach adulthood of the surviving fourth larvae both in the CAP and CCM treated groups became significantly longer compared to the controls. In addition, the body weight of newly emergent female and male adults in the two CAP agent treated groups was smaller. Except for the lower efficiency in pupation, including the developmental time, the emergence rate and adult (male and female) weight of the surviving individuals in the CCM treated groups was not significantly different from that of the pure CAP. 

### 3.5. Effect of the CCMs on the Reproduction of T. castaneum

**Figure 4:**
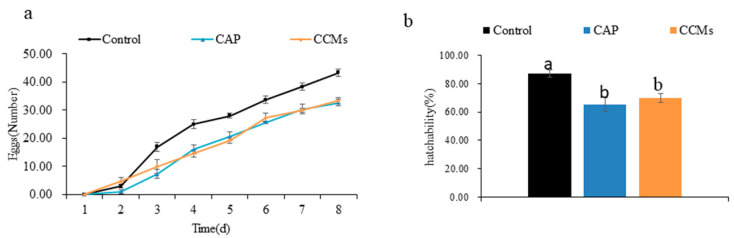
Effect of the CAP containing agents on the *T. castaneum* oviposition (**a**) and hatching (**b**). Data are expressed as mean ± SD (*n* = 3); values followed by different letters were significantly different by Tukey’s multiple range test. The one-way ANOVA result was df = 2, F = 20.394, *p* < 0.05.

The changes in the reproduction of the *T. castaneum* adults treated with CAP and CCMs were investigated (Figure 4). The number of eggs was decreased by nearly 30% in two treatments (Figure 4a), and the average hatching rate was decreased by 30.22% in the CCM treated groups and 34.64% in the CAP treated groups (Figure 4b). There was no significant difference between the CCM and CAP effectiveness on the *T. castaneum* egg-laying and hatching observed during the experiment.

## 4. Discussion

Capsaicin (CAP) is well-known for a variety of biological effects; a large number of studies have suggested its strong potential for application in insect pest control. However, its pungency, poor water solubility, and instability confine its practical application [16,18,35]. Microencapsulation is considered to be a useful method to solve these problems [35,36]. In this paper, capsaicin microcapsules (CCMs) were prepared by layer-by-layer self-assembly, using chitosan and carboxymethyl chitosan (CMCS) as shell materials. Further, its loading efficiency and physiochemical and insecticidal properties were carefully evaluated. The loading efficiency and release properties are important for evaluating the encapsulation quality [29]. In this paper, the loading efficiency of the CAP in the prepared CCMs was 64.31%, similar to previous reports [29,37,38]. This suggested good encapsulation of the CAP in the prepared microcapsules. The performance of the CAP slow-releasing microcapsules changed with the solutions’ pH values [37,39]. The analysis showed that the CAP loaded in the CCMs had the highest release efficiency in the alkaline solution (pH 9.94), followed by the acid solution (pH 3.03) and neutral solution (pH 7.0). Alongside the loaded CAP, the shell materials used to entrap the active ingredients might be another important factor affecting the microcapsules’ release characteristics. As is known, chitosan and its derivative are considered as promising encapsulating agents due to the presence of D-glucosamine in the structure and the positive charge on its amino groups [29]. Chitosan and CMS were selected as shell materials for the CAP microcapsules, giving the product pH sensitivity, high loading efficiency, and water solubility, as in previous reports [39,40,41,42]. The loaded CAP release from the CCMs in vitro were first promoted by swelling and diffusion, and the subsequent shell material degradation led to the sustained release of the loaded CAP from microcapsules [39]. Thus, the concentration of the loaded CAP release increased with exposure time. The loading efficiency of the CCMs was affected by various factors, such as the concentration of the CAP, the shell materials, the pH, the temperature, and the method used during preparation [39,42]. The next step is to investigate the influence of these factors on the insecticidal properties of CCMs. 

The analysis of the FTIR spectrum showed the interactions between the CAP and chitosan in the prepared CCMs. The FTIR spectra of the CCMs were similar to that of the pure CAP in peak positions, while its peak intensity was much weaker [36]. This suggested that there was an interaction between the CAP and the chitosan and its derivatives, disturbing the characteristic peak intensity. In the spectrum of pure capsaicin, the broad absorption peaks at 3293.43 cm^−1^ and 2926.43 cm^−1^ might be associated with the stretching of -OH bond and -C-H aliphatic stretching vibrations, respectively; the bands at 1641.95 corresponded to the stretching vibrations of C = O, the bands at 1524.60 cm^−1^ were the stretching of the polyphenol skeletal, and the bands at 1033.91 cm^−1^ were characteristic of the C-O-C stretching vibrational modes, which were the characteristic peaks of pure capsaicin [29,41,43,44]. The spectrum of the CCMs showed the increased O-H bond peak expanded and shifted to a higher number, compared to the pure CAP, which meant that the H bonds might form between the phenolic OH or C = O groups of capsaicin and the -OH or -NH_2_ groups of chitosan and its derivative; the intensity of the C-H aliphatic stretching vibrations increased might be positively correlated with the amount of capsaicin [45]. These changes confirmed the interaction between the CAP and chitosan molecules and the successful encapsulation of CAP. 

XRD analysis affirmed the V-type crystallites of CAP based on the strong reflections (2θ) at 12.080°, 20.467°, 24.152°, 25.162°, and 30.369°, as in previous reports [39]. Interestingly, the XRD graph of the CCMs showed similar diffraction Bragg angles peaks for the pure CAP, which suggested the high purity and fine crystalline of the prepared CCMs as expected. Furthermore, the crystalline form of the CCMs might be slightly different from that of the pure CAP due to the interaction between the CAP and the chitosan molecules [38]. However, the details of the interaction between the CAP and chitosan in the prepared CCMs were not clear.

The TEM image analysis revealed the difference between the pure CAP and the CCMs. Both the pure CAP and CCMs had a similar amorphous shape. This suggested that the structure of the CAP granules was not completely disrupted, and there was weak interaction between the CAP and chitosan molecules. A thick syrup layer may be formed by the wall materials (chitosan and CMCS) attaching to the surface of CAP, which made the CCMs particles more amorphous compared with that of the pure CAP agents; the particle size of the CCMs increased during the encapsulation process, and finally formed an irregular spherical shape [25,43]. The XRD and SEM analysis showed the particle size of CCMs was about 100 um, as previous studies suggested [38]. The particle sizes and morphologies of these microcapsules may be influenced by the preparation conditions (i.e., the CAP concentration, the chitosan/CAP ratio, the pH, and the drying methods), similar to other microcapsules [20,46]. In the future, we could further investigate the effects of these test parameters on the structural integrity of the CCMs, as well as the biochemical and physical characteristics (i.e., stability, release efficiency, and insecticidal property).

Various studies have revealed that capsaicin has lethal and antifeedant effects on various insect pests, which might serve as a potential insecticide for the protection of stored products [47,48]. Our research also proved that the capsaicin agent had high efficiency as a larvicide and adulticide in proper concentrations. The results showed that the dose of capsaicin and the efficiency against the *T. castaneum* adults were linearly dependent, as in previous reports [49]. Although the literature has revealed that CAP acted as an ovipositional repellent by disturbing the acid–base status, transient receptor potential vanilloid (TRPV) channel, oxidative innate immunity, intestinal dysplasia, and some critical enzymes (i.e., glutathione-S-transferase, Na^+^/K^+^-ATPase), the functional mechanism remains unclear [18,48,50].

Interestingly, the capsaicin microcapsules prepared showed a higher efficiency against *T. castaneum* than that of pure CAP. This suggested that the insecticidal efficacy of the CAP was enhanced by chitosan-based encapsulation. Chitosan and its derivatives (i.e., CMCS) could be used in pesticide delivery, copolymeric concentration, and nanomicellar, which could effectively prevent ecological harm, including the photodegradation of the active ingredients [51]. In addition, chitosan could give the CAP loaded in microcapsules good solubility and permeation into the insect gut, causing a series of adverse reactions, such as delayed growth and development, decreased adult weight and reproduction rate, and even death [49,52]. This experiment further suggested that the CAP could significantly disturb the growth and development of the insects. It was evident that even coated with shell materials (chitosan and CMCS), the concentration of the CCMs at the LC_50_ had high repellent activities against *T. castaneum,* just as with the pure CAP. 

Although there were no significant differences between the CCMs and CAP-treated groups, the CCMs are a better fit for stored product control due to their biocompatibility, low toxicity to the operator, high-release properties, and so on. At the same conditions, The KT_50_ of CCMs against *T. castaneum* was longer than that of CAP, suggesting the good slow–release performance of CCMs. According to the above analysis, the physical characteristics of chitosan and CMCS might be an important factor in the delivery of microcapsules into the insect gut; however, the details of their mechanism are still unknown.

Capsaicin’s function as an insect ovipositional repellent has been reported. The results showed that the CAP containing agents significantly affected the reproduction of *T. castaneum*, exhibiting the inhibition of oviposition and its offspring hatching. Previous studies have revealed that females were robustly repelled from laying eggs on CAP–containing sites, which might be mediated by their nociceptive neurons expressing the *painless* gene [18,47]. In addition, the structure of the capsaicin containing agent was another important factor affecting its ovipositional repellent activities [47]. In this study, the CCMs were amorphous in shape, and the structure–activity relations of the CCMs used as pesticides against stored product insects need to be further studied in future. The prepared CCMs showed high potential to be an alternative to traditional fumigants for stored product protection.

## 5. Conclusions

In conclusion, this study reported the preparation of CCMs using chitosan and carboxymethyl chitosan as shell materials. The prepared CCMs had a high loading efficiency and a good release property in acid and basic solutions, while they were stable in a neutral salt solution. The structural characteristics of the CCMs obtained by FTIR, XRD, and SEM showed the changes in the structure of the CAP after being encapsulated with shell materials. The FTIR and XRD analysis of the CAP microcapsules showed the interactions between the CAP and the shell materials (chitosan and carboxymethyl chitosan). It demonstrated that the CCMs particles had high crystallinity and a partially amorphous form, with an average size of 106.24 μm. 

In addition, the CCMs showed a significant effect on the survival, fecundity, hatching, and development of *T. castaneum*. The CCMs were more effective against the *T. castaneum* adult with an LC_50_ at 41.16 mg/kg, compared with the CAP (LC_50_ = 44.67 mg/kg). The use of the CCMs not only affected the insect growth and development but also reproduction. The growth period of *T. castaneum* was significantly prolonged, and the number of eggs laid, pupation rate, emergence rate, and hatching rate were significantly decreased in the CCM and CAP treated groups, while there were some significant differences between the two treatments. This study provides a favorable method for CAP to be prepared into microcapsules for controlling stored grain pests. Further studies on the correlation with its main property parameters as a biopesticide are needed for the rational design of microcapsules with improved bioactivities. 

## Figures and Tables

**Table 1:** Effects of the CCMs and CAP on *T. castaneum* larval development.

Processing Mode	Development Time of Fourth Larva to Adult Instar (Days)	Pupation Rate (%)	Emergence Rate (%)	Adult Weight (mg)	
				Male	Female
Control	4.78 ± 0.20 a	91.3 ± 0.90 c	94.2 ± 0.50 b	2.263 ± 0.012 b	2.364 ± 0.011 b
CAP	7.41 ± 0.20 b	65.33 ± 0.88 a	66.38 ± 0.60 a	2.038 ± 0.011 a	2.124 ± 0.015 a
CCMs	7.59 ± 0.195 b	69.6 ± 0.60 b	65.73 ± 0.62 a	2.029 ± 0.010 a	2.142 ± 0.013 a

Note: Data were expressed as mean SD (*n* = 3); those followed by different letters were significantly different by Tukey’s multiple range test. The one-way ANOVA results for the development time, pupation rate, emergence rate, and male and female weight were df_t_ = 2, df_e_ = 6, F = 196.018, *p* < 0.001; df_t_ = 2, df_e_ = 6, F = 1021.69, *p* < 0.001; df_t_ = 2, df_e_ = 6, F = 2532.34, *p* < 0.001; df_t_ = 2, df_e_ = 6 F = 410.734, *p* < 0.001; df_t_ = 2, df_e_ = 6 F = 35.515, *p* < 0.001, respectively.

## Data Availability

Data is contained within the article.

## Supplementary Materials

The following supporting information can be downloaded at: https://www.mdpi.com/article/10.3390/insects14010027/s1, Figure S1: The release of pure capsaicin in various solutions at different pH values (a) 50% ethanol solution, y = 0.366 x − 0.3021, R^2^ = 0.9963; (b) NaOH solution (pH 9.94), y = 0.0124 x + 0.0254, R² = 0.9983; (c) acetate solution (pH 3.03) y = 0.016 x + 0.0379, R² = 0.9979; (d) NaCl solution (pH 9.94),y = 0.0126 x + 0.032, R² = 0.9973, Table S1: Mortality of T. castaneum adults exposed to different concentrations of pure CAP and CCMs, Table S2. Mortality of *T. castaneum* adults exposed to 30mg/Kg of pure CAP and CCMs for different time.
