# Supplementary material for: Microencapsulation of Capsaicin in Chitosan Microcapsules: Characterization, Release Behavior, and Pesticidal Properties against Tribolium castaneum (Herbst)
Source: Insects. 2022 Dec 27;14(1):27. doi: 10.3390/insects14010027 (PMC9864733; doi:10.3390/insects14010027)
Supplement: Supplementary file 1 [file insects-14-00027-s001.zip › insects-2094244-supplementary.pdf]

Supporting information

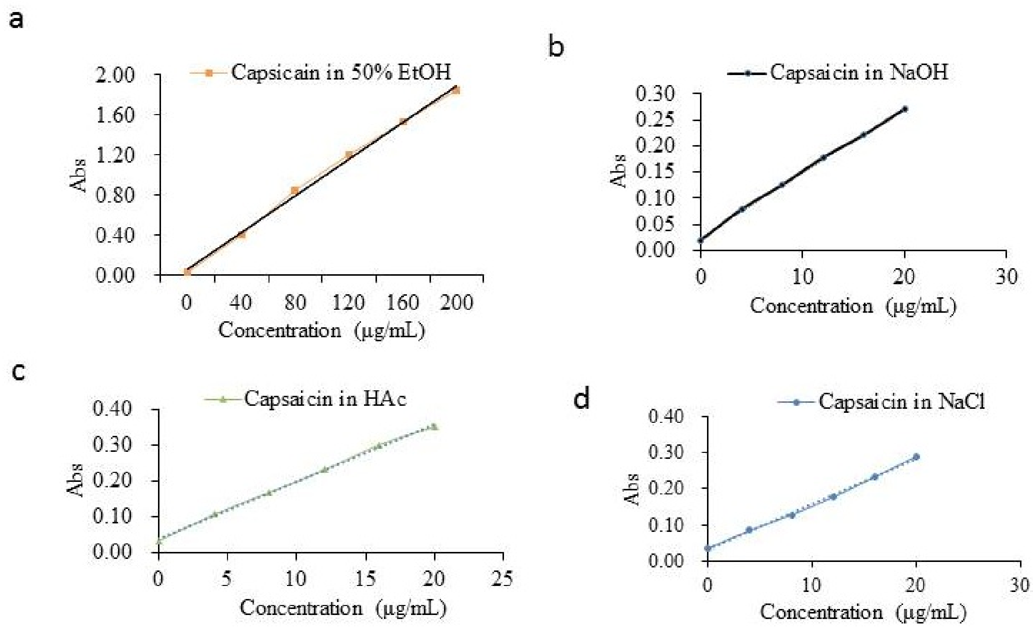

Fig. S1. The release of pure capsaicin in various solutions at different pH values

(a) 50% ethanol solution, $y = 0.366x-0.3021$, $R^2 = 0.9963$; (b)NaOH solution (pH 9.94), $y = 0.0124x + 0.0254$,$R^2 = 0.9983$; (c) acetate solution (pH 3.03) $y = 0.016x + 0.0379$,$R^2 = 0.9979$; (d) NaCl solution (pH 9.94),$y = 0.0126x + 0.032$, $R^2 = 0.9973$.

Table 1S Mortality of *T. castaneum* adults exposed to different concentrations of pure CAP and CCMs

**Pesticide**	**Con(mg/ kg flour)**	**7 days Mortality (%)± SD**	**Slope function(S)**	**LC_{50}±SE (mg/kg)**	**LCL-UCL (mg/kg)**	**$\chi2$**
CAP	Control	6.67±0.58a	1.196±0.335	31.37	22.92-48.54	5.292*
	10	28.57±1.53b				
	20	37.50±0.58bc				
	30	53.57±1.15d				
	40	51.79±2.00de				
	50	60.71±1.53e				
CCMs	Control	6.67±0.58a	1.389±0.34	29.75	22.59-41.19	6.237*
	10	28.57±1.15b				
	20	37.50±1.53bc				
	30	48.21±1.53bcd				
	40	53.57±2.08cd				
	50	67.86±1.00e				

Means followed by different letters are significantly different ($P < 0.05$; one-way ANOVA and Tukey's test). SD, standard deviation; SE, standard error; Con, concentration.LCL, lower confidence limits, UCL, upper confidence limits, $\chi2$, Chi-square test.

Table 2S Mortality of *T. castaneum* adults exposed to 30mg/Kg of pure CAP and CCMs for different time

**Pesticide**	**Exposure time(d)**	**Mortality (%)± SD**	**Slope function(S)**	**KT_{50}±SE (d)**	**LCL-UCL (d)**	**$\chi 2$**
CAP	0	0.00	2.404±0.312	5.86	5.10-7.08	13.26*
	1	5.00±1.00a				
	2	10.00±1.73a				
	3	20.34±0.58ab				
	4	35.59±1.53bc				
	5	43.10±2.00c				
	6	49.12±2.52c				
	7	53.57±1.15c				
CCMs	0	0.00	1.442±0.269	8.994	6.70-15.74	7.372*
	1	11.67±0.58a				
	2	11.67±0.58b				
	3	20.34±0.58bc				
	4	23.73±1.00bc				
	5	29.31±1.53cd				
	6	38.60±1.53de				
	7	48.21±1.53e				

Means followed by different letters are significantly different ($P < 0.05$; one-way ANOVA and Tukey's test). SD, standard deviation; SE, standard error; Con, concentration.LCL,lower confidence limits, UCL, upper confidence limits, $\chi 2$, Chi-square test.
